# Isolated goat and sheep milk allergy in an adult in the absence of bovine sensitization

**DOI:** 10.1186/s13223-025-00996-x

**Published:** 2025-12-01

**Authors:** Michael Aw, Gordon Sussman

**Affiliations:** 1 Department of Internal Medicine, Faculty of Medicine, McGill University Health Centre (MUHC), 1001 Decarie Boulevard, Montreal, QC H4A 3J1 Canada; 2 Division of Allergy & Clinical Immunology, University of Toronto, Toronto, ON Canada

**Keywords:** Milk protein allergy, Anaphylaxis, Case report

## Abstract

Cow’s milk (CM) allergy is the most common food allergy in children, with cross-reactivity to goat and sheep milk (GSM) representing a common outcome due to high protein homology. However, isolated GSM allergy in CM-tolerant individuals is rare and poorly documented in the literature. We describe a 27-year-old male of Mediterranean origin who experienced two episodes of anaphylaxis after consuming sheep and goat cheese, despite regular CM consumption. Skin prick testing confirmed sensitization to GSM but not CM. This report contributes to the growing evidence of adult-onset isolated GSM allergy. Isolated GSM allergy typically presents later in life and may be underrecognized due to limited clinical awareness. We review the potential for species-specific mammalian milk protein allergy, likely mediated by structural differences in casein proteins. Clinicians should be cognisant to consider GSM sensitization in patients presenting with allergic reactions to dairy products, especially in multicultural populations with increasing GSM dietary exposure.

## Intro

Cow’s milk (CM) allergy is the most common allergy in young children affecting up to 3% of the pediatric population [2]. CM allergy manifests through IgE and non-IgE mechanisms with most children developing immunologic tolerance by adolescence. Similarly, other mammalians milks such goat and sheep [1] share generally > 88% protein homology with CM resulting in frequent species cross-reactivity [2]. Approximately 90% of patients with GSM allergy are concurrently CM sensitized [3]. However, isolated GSM allergy in the absence of CM hypersensitivity is rare and our current understanding is limited to case report and case series data. We present a case of a 27-year-old male who experienced anaphylaxis following consumption of GSM cheese who was otherwise CM tolerant.

## Case

A 27-year-old male of Mediterranean origin was referred for allergist evaluation for a suspected sesame allergy. He was known for allergic rhinitis with sensitizations to house dust mite, birch, ragweed, cat, dog and Alternaria mould. He had no other medical history and did not use regular medication or consume recreational drugs. The patient had consumed a meal with cheese and black sesame and subsequently developed immediate severe nausea, throat tightness, dyspnea and diffuse urticaria. He was subsequently treated with intramuscular epinephrine in the emergency room.

The patient underwent skin prick testing (SPT) with appropriate histamine and 0.9% saline buffer control responses that was negative to fresh sesame and sesame oil. His serum sesame IgE was low reactivity (1.01 KU/L, normal range < 0.35 KU/L) and baseline tryptase 8.4 mcg/L. He had elevated total serum IgE (1167 KU/L) and peripheral eosinophilia (0.5 × 10^9^ cells/L), with the remainder of his blood count and biochemistry unremarkable. On a subsequent visit, he successfully completed a 5-step oral challenge to sesame and re-introduced sesame into his diet.

**Fig. 1:**
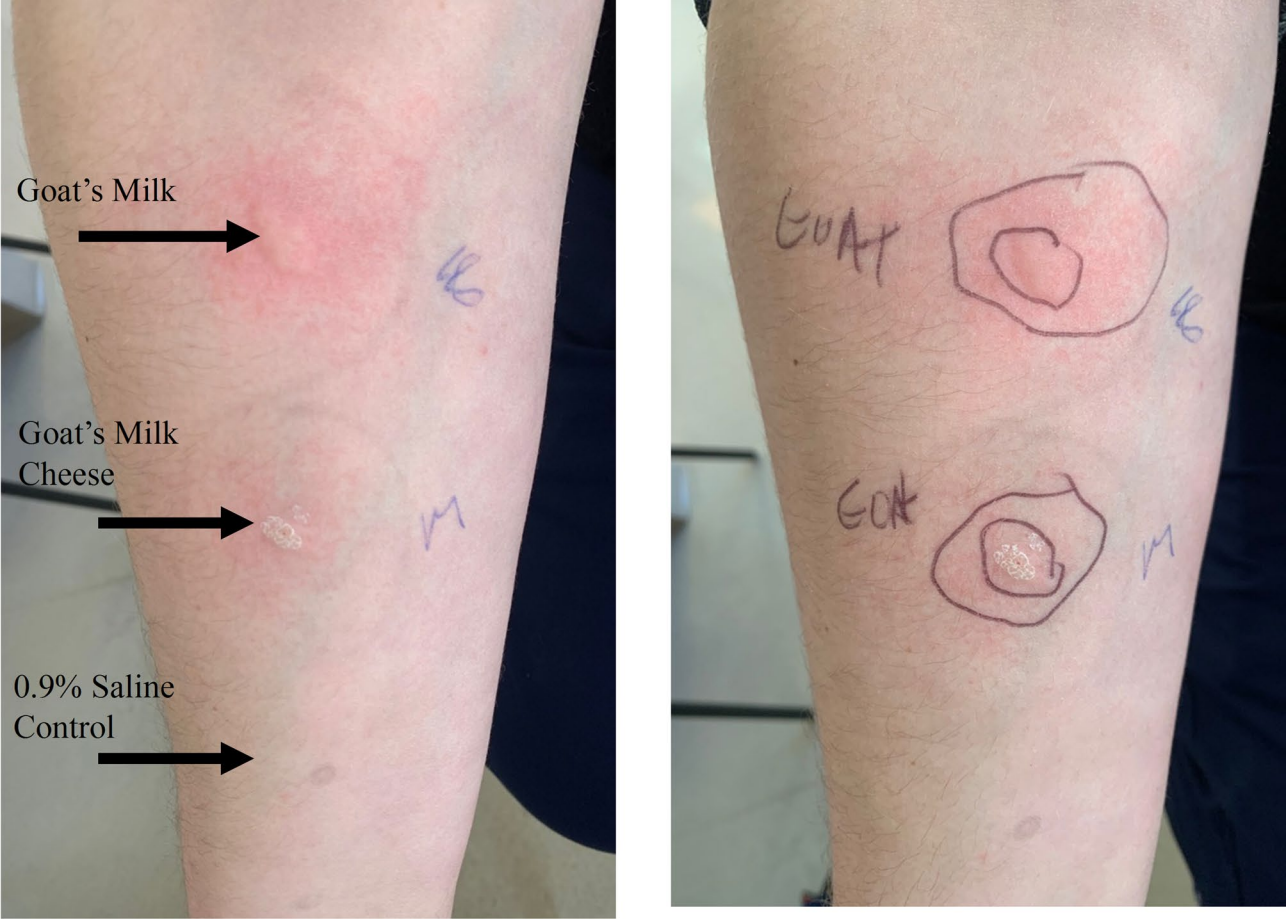
Highly positive skin prick test to goat milk and goat milk cheese (18 mm and 10 mm weal diameter compared with the negative control, respectively)

The patient returned to clinic with a second anaphylactic reaction manifesting as diffuse urticaria, severe gastrointestinal upset, throat tightness and dyspnea within 30 min of consuming sheep’s milk cheese. His SPT demonstrated a positive wheal (16 mm) to the suspected culprit sheep’s milk cheese and negative to CM, egg, shellfish (clam, lobster, scallop, shrimp, crab), wheat, rice, peanut and tree nuts (almond, pecan, walnut, cashew, hazelnut, brazil nut). Six months later, the patient experienced another anaphylactic episode with full body urticaria, dyspnea, vomiting and oropharyngeal pruritus and swelling while consuming goat’s milk cheese. SPT was performed against goat’s milk cheese (10 mm) and fresh goat milk (18 mm) which was positive (Fig. 1). Notably, the patient regularly consumed CM products including milk, cheese, and baked goods. He had previously consumed GSM cheese many years ago without issue.

The patient was advised to strictly avoid GSM with continued ingestion of CM containing products. An epinephrine autoinjector was also prescribed. Oral food challenges were not performed, as the history of a severe allergic reaction in conjunction with positive SPT results was considered adequate to establish a diagnosis of GSM allergy.

## Discussion

This case highlights the importance of recognizing rare food allergens. Specifically, clinicians should consider species-specific mammalian protein sensitization when evaluating patients with milk protein hypersensitivities.

Bovidae (family of cloven-hoofed ruminant mammals which includes cow, goat and sheep) milk consists of casein and whey. The homology of these proteins between CM and GSM exceeds 88% [2]. Specifically, casein which encompasses αS1, αS2, β, κ, and γ casein, are intrinsically unstructured proteins without clear tertiary structures, they are not easily denatured by heat and maintain their allergenicity [4]. Consequently, caseins are the prevalent allergen in cheese and baked milk products and are associated with prolonged sensitization. Immunoblotting studies have confirmed caseins as the most common allergen in isolated GSM allergy, although α-Lactalbumin sensitization with goat cheese has been reported [3, 5–8]. Hazebrouck et al. demonstrated a goat β-casein epitope differing by only five amino acids failed to cross-react with CM β-casein to degranulate cells passively sensitized with IgE antibodies from goat milk-allergic/CM-tolerant individuals [4]. This supports the importance of casein allergens and minimal structural variations in mediating species-specific allergic responses.

Currently, there are limited reports of isolated GSM allergy in adults (Table 1). Unlike CM allergy which typically presents in infancy or early childhood, GSM allergy often manifests later in life, particularly among older children and adults [3, 5–9]. CM can trigger both IgE-mediated and non-IgE-mediated reactions, whereas GSM allergy appears to be limited to immediate IgE-mediated responses [2]. However, the true incidence may be underestimated, as non-IgE-mediated conditions such as food protein-induced enterocolitis syndrome are challenging to confirm. The demographic of those with isolated GSM are frequently of European descent [3, 6, 8, 9]. This may reflect the dietary patterns of European populations, where GSM-derived products like yogurt and cheese are commonly consumed.

**Table 1:** Summary of adult and pediatric cases of isolated CGM allergy

Patient demographics	Country of origin	Co-morbidities	Allergen	Symptoms	SPT	SIgE^*^	Immunoblotting^#^	Reference
25 F	Japan	Atopic dermatitis, asthma	SC	Angioedema, gastrointestinal upset	GM + , SM + SC + , CM-	SM + CM-	α casein	Suzuki et al. [5]
25 M	Italy	nil	GC, SC	urticaria, angioedema, dyspnea	CM-,GM +	CM-, GM + , SM +	n/a	Wüthrich &Johansson [9]
27 F	Portugal	asthma, allergic rhinitis	GC	urticaria	GM + , GC + , CM-	GM-, CM-	α-Lactalbumin	Tavares et al. [8]
28 M	Canada	Allergic rhinitis	SC, GC	urticaria, gastrointestinal upset, dyspnea	SC + , GM + , GC + , CM-	N/A	N/A	**
39 M	Switzerland	Allergic rhinitis	GC, SC	Urticaria, angioedema, hypotension	SM + , SC + , GM + , GC + , CM-	SM + , GM +	κ-casein	Hirsiger et al. [3]
43 M	Spain	Nil	SM, SC	dyspnea, gastrointestinal upset, hypotension	GM + , SM + , SC + , CM-	CM-, GM + , SM +	α or β casein	Alvarez & Lombardero [6]
55 F	Australia	Atopic dermatitis, asthma	GC	Urticaria, dyspnea, angioedema	N/A	GM +	β & γ casein	Voskamp et al. [7]
54 M	Spain	N/A	GM & SM ***	Not specified	GC + SM + CM +	SM + GM- CM-	Casein not specified	Vinas et al. [11]
61 F	Spain	N/A	Mixed cheeses	Angioedema	GC + SC + CC-	GM- SM- CM-	N/A	Vinas et al. [11]
22 M	France	Dermatitis, asthma	GC	Urticaria, bronchospasm, anaphylaxis	N/A	GM + SM + CM-	N/A	Lamblin et al. [12]
52 F	France	Nil	GC	Urticaria, bronchospasm, anaphylaxis	GM + GC + SC + CC -	GM + SM + CM-	N/A	Lamblin et al. [12]
10 M	France	Nil	GM ***	Angioedema, urticaria	GM + CM +	GM + SM + CM +	N/A	Lamblin et al. [12]
11 F	France	Atopic dermatitis	GC ***	Angioedema	GC + SM + CM +	GM + SM + CM +	N/A	Attou et al. [13]
1–10 (n = 18, 4/18 F)	France	16/18 atopic dermatitis 9/18 asthma	GC	Common symptoms: angioedema, urticaria, oral pruritus	GC + 18/18) SC (14/18) CM- (18/18)	GM + (16/18) SM + (13/18)	N/A	Paty et al. [14]
1–16 (n = 28, 8/28 F)	France	19/28 asthma 14/28 atopic dermatitis 11/28 rhinitis	GC or SC	Common symptoms: angioedema, urticaria, asthma	GM + (28/28) SM + (17/28)	GM + (27/28) SM + (27/28) CM + (9/28)	α S1-, α S2- and β-casein	Ah-Leung et al. [15]
5 M	Italy	Atopic dermatitis, asthma, rhinitis	SC	Urticaria, oropharyngeal pruritus, rhinorrhea	GM + GC + SM + SC + CM-	CM- CC-	N/A	Calvani and Alessandri [16]
6 M	China	Atopic dermatitis, allergic rhinitis, shellfish allergy	GM	Urticaria, bronchospasm	CM +	CM + CM-	N/A	Goh et al. [17]
5 M	America	Asthma, allergic rhinitis	GM SM	Urticaria, bronchospasm, vomiting	GM + SM + CM +	GM + SM + CM +	N/A	Pham and Wang [18]
9 M	Portugal	N/A	GC SC	Angioedema, dizziness	SM + GM + CM-	GM + CM-	N/A	Martins et al. [19]

Abbreviations: F, female; M, male; CM, cow’s milk; SM, sheep’s milk; SC, sheep’s cheese; GM, goat’s milk; GC, goat’s cheese; CC, cow’s cheese

*Measured by serum specific IgE or radioallergosorbent test, **Current case report, ^#^Western blot or immunoblotting. ***Patient tolerates cow’s milk products though sensitized

In our case, the patient developed symptoms in his twenties which is consistent with the typical age range for adult-onset GSM allergy (20–40 years) [3, 5, 6, 8, 9]. Although he followed a primarily North American diet, the patient was of Mediterranean ancestry which could have increased his GSM exposure risk. Despite anticipated dietary exposure to GSM products among Canadians of European heritage, isolated GSM allergy remains rarely reported. To our knowledge, this represents the only documented adult case of isolated GSM allergy in Canada [10].

Sensitization to both goat and sheep’s milk, as seen in the present patient, occurs in approximately half of cases, reflecting the high antigenic similarity between these proteins [3, 5, 6]. Therefore, individuals with a suspected GSM allergy should be evaluated for sensitization to all three species. Moreover, the presence of isolated GSM allergy is relevant for patient’s undergoing CM oral immunotherapy (OIT) since the desensitization may not confer tolerance to non-bovine species. Notably, 25% of children who successfully complete CM OIT demonstrate GSM allergy [1]. Further studies are warranted to estimate the true prevalence of isolated GSM allergy and clarify risk factors and demographic patterns associated with GSM sensitization.

This case adds to the limited literature on isolated GSM allergy in adults and underscores the importance of considering species-specific sensitization in those with suspected dairy allergies, particularly in adult patients. Given the increasing dietary diversity associated with multicultural populations in North America, clinicians should recognize this this rare allergic phenotype that may be encountered more frequently in clinical practice.

## Data Availability

No datasets were generated or analysed during the current study.

## Abbreviations

CM: Cow’s milk
GSM: Goat and sheep’s milk
SM: Sheep’s milk
SC: Sheep’s cheese
GM: Goat’s milk
GC: Goat’s cheese
SPT: Skin prick testing
OIT: Oral immunotherapy

## Declarations

### Clinical implications

Isolated goat and sheep milk allergy can occur despite cow’s milk tolerance, highlighting the need for species-specific evaluation in patients with dairy-induced anaphylaxis.
